# Psilocin, the Psychoactive Metabolite of Psilocybin, Modulates Select Neuroimmune Functions of Microglial Cells in a 5-HT_2_ Receptor-Dependent Manner

**DOI:** 10.3390/molecules29215084

**Published:** 2024-10-28

**Authors:** Kennedy R. Wiens, Noah A. H. Brooks, Ishvin Riar, Bridget K. Greuel, Ivan A. Lindhout, Andis Klegeris

**Affiliations:** Laboratory of Cellular and Molecular Pharmacology, Department of Biology, University of British Columbia Okanagan Campus, Kelowna, BC V1V 1V7, Canada

**Keywords:** affective disorders, depression, 5-hydroxytryptamine, neurodegenerative diseases, neuroprotection, phagocytic activity, reactive oxygen species, reactive nitrogen species, serotonin

## Abstract

Neuroinflammation that is caused by microglia, the main immune cells of the brain, contributes to neurodegenerative diseases. Psychedelics, including psilocybin and lysergic acid diethylamide (LSD), possess certain anti-inflammatory properties and, therefore, should be considered as drug candidates for treating neuroinflammatory pathologies. When ingested, psilocybin is rapidly dephosphorylated to yield psilocin, which crosses the blood–brain barrier and exerts psychotropic activity by interacting with the 5-hydroxytryptamine 2A receptors (5-HT_2A_Rs) on neurons. Since microglia express all three 5-HT_2_R isoforms, we hypothesized that, by interacting with these receptors, psilocin beneficially modulates select neuroimmune functions of microglia. We used microglia-like cell lines to demonstrate that psilocin, at non-toxic concentrations, did not affect the secretion of tumor necrosis factor (TNF) by immune-stimulated microglial cells, but significantly inhibited their phagocytic activity, the release of reactive oxygen species (ROS), and nitric oxide (NO) production. The inhibitory activity of psilocin on the latter two functions was similar to that of two selective 5-HT_2_R agonists, namely, 25I-NBOH and Ro60-0175. The role of this subfamily of receptors was further demonstrated by the application of 5-HT_2_R antagonists cyproheptadine and risperidone. Psilocin should be considered a novel drug candidate that might be effective in treating neuroimmune disorders, such as neurodegenerative diseases, where reactive microglia are significant contributors.

## 1. Introduction

While the aggregation of amyloid β and the hyperphosphorylation of tau play well-established roles in the pathophysiology of Alzheimer’s disease (AD), the involvement of persistent neuroinflammation in the development and progression of this and other neurodegenerative disorders is increasingly accepted [1]. Neuronal death and neuroinflammatory responses in AD are accompanied by the chronic overactivation of glial cells, particularly microglia [2,3]. When confronted with abnormal protein aggregates, microglia facilitate the innate neuroimmune response. In part, this involves the clearance of harmful particles by phagocytosis and the release of cytokines, such as the pro-inflammatory tumor necrosis factor (TNF), and cytotoxins, including reactive oxygen species (ROS) and nitric oxide (NO) [4]. While these functions are essential for mounting an effective acute immune response, neurodegeneration, once triggered, leads to the chronic adverse reactivity of microglia. This causes the sustained secretion of pro-inflammatory mediators and excess phagoptosis, contributing to neuronal death [5,6]; therefore, anti-inflammatory therapies aimed at ameliorating reactive microglia represent feasible options in the search for effective treatments for these disorders [7,8].

Anti-inflammatory drugs are a class of medication aimed at suppressing or modulating inflammatory responses. The long-term use of conventional non-steroidal anti-inflammatory drugs (NSAIDs) has previously been associated with a decreased risk of AD in epidemiological studies [9]; however, conflicting results have been observed in clinical trials [10]. Additionally, several other commonly used inflammation-reducing therapies, such as corticosteroids, have proven to lack protective effects in AD treatment [11]; therefore, novel molecular targets and drug candidates that can be used to effectively suppress neuroinflammation in a broad range of neurodegenerative diseases need to be identified. Anti-inflammatory drugs often exert their effects by reducing the activity of enzymes and transcription factors, such as cyclooxygenase (COX)-2, nicotinamide adenine dinucleotide phosphate (NADPH)-dependent oxidase (NOX), inducible NO synthase (iNOS), and nuclear factor (NF)-κB. Such inhibitory activities diminish the production of inflammation-inducing and -propagating prostaglandins, ROS, NO, and cytokines [12,13,14,15]. Psychedelics, including psilocybin, mescaline, and lysergic acid diethylamide (LSD), have recently been shown to possess anti-inflammatory properties [16]. Since this heterogenous group of compounds remains relatively unexplored in the context of neuroinflammation and neurodegenerative diseases, they may possess untapped potential as drug candidates for the treatment of these disorders [17].

Depression is often associated with alterations in 5-hydroxytryptamine (5-HT, serotonin) neurotransmission. Most first-line antidepressants modulate specific aspects of 5-HT signaling, such as its reuptake, but there are treatment-resistant cases that do not respond to such therapies [18]. Notably, increasing evidence indicates that neuroinflammation also contributes to the pathogenesis of depression [19]. Treatment-resistant depression cases in particular are associated with increased TNF concentrations [20]. In addition, higher-than-normal plasma concentrations of both ROS and NO have been detected in individuals experiencing depressive symptoms [21,22]. Recently, antidepressant drugs, such as selective serotonin reuptake inhibitors (SSRIs), have been shown to reduce neuroinflammation [23]. Therefore, drugs with antidepressant activity that are also confirmed to cross the blood–brain barrier should be considered for use to suppress neuroinflammation in neurodegenerative diseases [24,25]. 

Psilocin (4-hydroxy-N,N-dimethyltryptamine) is the bioactive metabolite of the naturally occurring psychedelic alkaloid psilocybin, which was first isolated from mushrooms of the *Psilocybe* genus [26]. Since their discovery, methods of synthetic production for both psilocin and psilocybin have been developed. When ingested, psilocybin undergoes rapid dephosphorylation by alkaline phosphatase in the small intestine, kidneys, and plasma to yield psilocin, which freely crosses the blood–brain barrier and exerts psychotropic activity [27]. The distinctive psychedelic effects induced by psilocin result from altered serotonin signaling, primarily due to the activation of the 5-HT_2A_ receptor (5-HT_2A_R) isoform on frontal cortex pyramidal neurons [28,29]. Likely due to this stimulatory activity of its main metabolite, psilocybin has emerged as a promising novel antidepressant, as shown in multiple human clinical trials, significantly alleviating symptoms in various forms of depression, including in treatment-resistant cases [30,31,32]. Recently, it has been proposed that the clinically meaningful antidepressant activity of psilocin could be due in part to its anti-inflammatory effects, which are also mediated by 5-HTRs expressed by neurons and glial cells [16,33]. Therefore, we hypothesized that the anti-neuroinflammatory activity of psilocin is mediated by its activation of 5-HT_2_R isoforms expressed by microglia, thereby downregulating their phagocytic activity and reducing their secretion of cytotoxins. 

To test this hypothesis, in addition to psilocin, we employed two established ligands of 5-HT_2_Rs, namely, 25I-NBOH and Ro60-0175. All three compounds exhibit unique binding patterns with regard to 5-HTRs [34,35]. Psilocin displays a high level of affinity for the 5-HT_2A_R but is also capable of binding to the 5-HT_2B_R, 5-HT_2C_R, and 5-HT_1A_R isoforms, albeit with a lower level of affinity [36,37]. 25I-NBOH demonstrates a high level of affinity for the 5-HT_2A_R and, in contrast to psilocin, is highly selective for this receptor isoform in relation to other members of the 5-HT_2_R subfamily [38]. Conversely, Ro60-0175 has a higher level of affinity towards the 5-HT_2C_R isoform than 5-HT_2A_ [39]. In this study, we found that psilocin significantly inhibited phagocytic activity and the production of ROS and NO by immune-activated microglial cells. We also demonstrated that the inhibitory activity of psilocin on ROS and NO production by microglial cells was similar to that of the two established 5-HT_2_R agonists, 25I-NBOH and Ro60-0175. Additionally, the 5-HT_2_R antagonists cyproheptadine and risperidone were employed to provide further evidence that the identified modulatory effects of psilocin were 5-HT_2_R-dependent. 

## 2. Results

### 2.1. Effects of Psilocin on Phagocytosis of Latex Beads and Secretion of Tumor Necrosis Factor by BV-2 Murine Microglia

**Figure 1:**
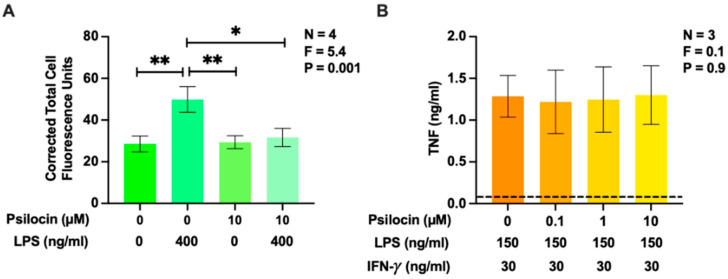
The effect of psilocin on the phagocytosis of latex beads and the secretion of TNF by BV-2 murine microglia. Varying concentrations of psilocin, shown on the abscissa, were administered 20 min prior to the addition of LPS (**A**) and LPS plus IFN-γ (**B**). After a 24 h incubation period, cells were exposed to fluorescent latex beads for 1 h and corrected total cell fluorescence values, taken from 189 to 192 randomly selected cells per experimental condition, were measured in a blinded manner (**A**). In a separate experiment, TNF concentration in cell-free supernatants was quantified after 24 h of exposure to stimuli. The dashed line represents the detection limit of the enzyme-linked immunosorbent assay (ELISA) (**B**). Data from 3 to 4 experiments, completed on different days, are shown as means ± SEM. The *p* and F values displayed in the figures were calculated using one-way (**A**) or randomized block one-way (**B**) ANOVA. * *p* < 0.05 and ** *p* < 0.01, as determined according to Tukey’s post hoc test.

**Figure A1:**
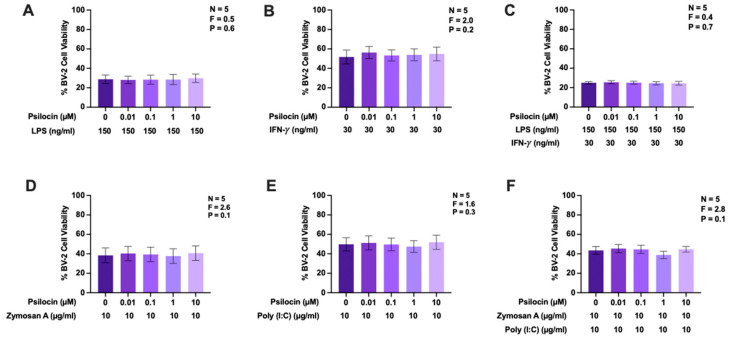
Effects of psilocin on viability of BV-2 murine microglia exposed to diverse stimuli. Varying concentrations of psilocin were administered 20 min prior to the addition of four different stimuli or their combinations as displayed in the figure panels (**A**–**F)**. Following a 24 h incubation period, viability of cells was assessed by the MTT assay. Data from five independent experiments completed on different days are shown as means ± SEM. *p* and F values displayed in the figures were calculated using randomized block one-way ANOVA.

The phagocytic activity of primary murine microglia has been shown to decrease in response to treatment with the 5-HT_2_R agonists 5-HT and 2,5-dimethoxy-4-iodoamphetamine (DOI) [40]. In a more recent investigation, Kozlowska et al. [41] showed that psilocin downregulates the phagocytosis of viable neurons by primary murine CD11b+ microglia. Additionally, psilocin has been demonstrated to inhibit TNF secretion by RAW 264.7 murine macrophages [42] and exposure to psilocybin reduces TNF levels in mouse brains and human sera [33,43]; however, the opposite effect has been reported on serum TNF levels in rats [44]. Therefore, we first investigated whether psilocin inhibited the phagocytic activity and TNF secretion of immune-stimulated BV-2 murine microglia. The bacterial endotoxin lipopolysaccharide (LPS) and the combination of LPS plus interferon (IFN)-γ were employed as known inducers of phagocytic activity and cytokine secretion in multiple cell types, including BV-2 cells [45,46]. Psilocin was used at up to the maximum non-toxic concentration (10 µM), which was determined in preliminary experiments. The phagocytic activity of BV-2 cells was nearly doubled after their incubation with LPS for 24 h (Figure 1A). On its own, psilocin did not affect this function but completely inhibited the stimulatory effect of LPS. This effect was not caused by the cytotoxicity of psilocin since, at the 0.01 to 10 μM range, it did not inhibit TNF secretion by BV-2 cells (Figure 1B). The lack of toxic effects of psilocin towards immune-stimulated BV-2 cells at this concentration range was also confirmed using the 3-(4,5-dimethylthiazol-2-yl)-2,5-diphenyl tetrazolium bromide (MTT) assay (Figure A1).

### 2.2. Effects of Psilocin, 25I-NBOH, and Ro60-0175 on Reactive Oxygen Species Generation by HL-60 Human Microglia-Like Cells

Nkadimeng et al. [47] report that the use of extracts from psilocybin-containing mushrooms decreases intracellular ROS levels in cardiomyocytes. To the best of our knowledge, the effects of psilocin or psilocybin on the generation of ROS by primary microglia or microglial model cells have not been determined. To investigate the effects of psilocin on ROS production in microglia-like cells, we used differentiated human HL-60 myelomonocytes, which respond similarly to microglia with regard to the respiratory burst when they are primed with LPS and subsequently stimulated with the bacterial peptide N-formyl-Met-Leu-Phe (fMLP) [48,49,50]. While psilocin has a high affinity for 5-HT_2_Rs, it also binds to other 5-HTR isoforms. Therefore, in addition to psilocin, we studied the effects of two other 5-HT_2_R ligands on the respiratory burst response: the specific 5-HT_2A_R agonists 25I-NBOH and Ro60-0175. The latter is more selective towards 5-HT_2C_R than 5-HT_2A_R. We hypothesized that psilocin would display a level of biological activity comparable to that of these established specific agonists when applied to microglial 5-HT_2_Rs. All three drugs were used at or below the maximum non-toxic concentrations determined in the preliminary experiments. 

**Figure 2:**
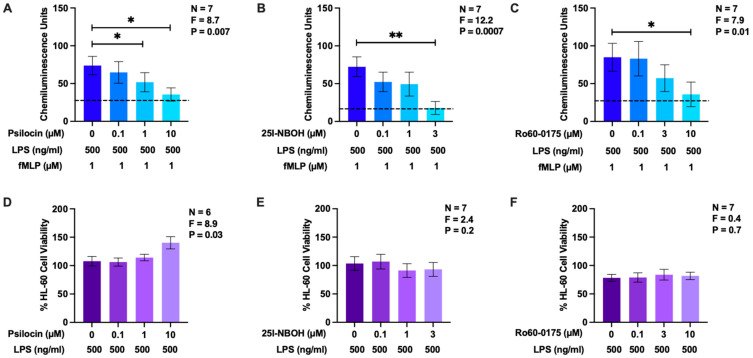
The effects of psilocin, 25I-NBOH, and Ro60-0175 on ROS generation by HL-60 human microglia-like cells and their viability. Varying concentrations of psilocin, 25I-NBOH, or Ro60-0175, shown on the abscissa, were added to differentiated HL-60 cells 20 min prior to their priming with LPS for 24 h. The respiratory burst response was induced by fMLP, and CHL was measured to quantify ROS production (**A**–**C**). The viability of HL-60 cells in separate wells was measured by the MTT assay (**D**–**F**). The dashed line represents the mean baseline CHL signal from unprimed but fMLP-stimulated cells. Data from 6 to 7 experiments completed on different days are shown as means ± SEM. *p* and F values displayed in the figures were calculated using randomized block one-way ANOVA. * *p* < 0.05 and ** *p* < 0.01 according to Dunnett’s post hoc test.

In comparison to the unprimed cells, LPS priming upregulated ROS production by HL-60 cells in response to fMLP stimulation (Figure 2A–C). Psilocin (1 and 10 µM), 25I-NBOH (3 µM), and Ro60-0175 (10 µM) significantly decreased the production of ROS by LPS-primed and fMLP-stimulated HL-60 cells. 25I-NBOH was the most effective drug, inhibiting approximately 75% of the maximal CHL signal (Figure 2B), followed by Ro60-0175 (Figure 2C) and psilocin (Figure 2A), which caused 58% and 52% reductions, respectively. At the concentrations tested, psilocin, 25I-NBOH, and Ro60-0175 did not significantly decrease the viability of HL-60 human cells (Figure 2D–F). This confirmed that the observed inhibitory effects of these drugs on ROS production were not due to the induction of HL-60 cell death.

### 2.3. Effects of Psilocin, 25I-NBOH, and Ro60-0175 on Nitric Oxide Production by BV-2 Murine Microglia Cells 

**Figure 3:**
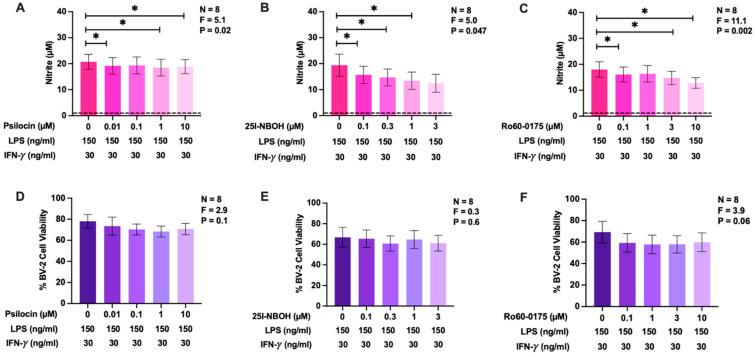
The effects of psilocin, 25I-NBOH, and Ro60-0175 on NO production and the viability of BV-2 murine microglia. Varying concentrations of psilocin, 25I-NBOH, or Ro60-0175, shown on the abscissa, were added to BV-2 cells 20 min prior to stimulation with LPS plus IFN-γ. Following a 24 h incubation period, nitrite in cell-free supernatants was quantified by the Griess assay (**A**–**C**) and cell viability was measured by the MTT assay (**D**–**F**). The dashed line represents the detection limit of the Griess assay (**A**–**C**). Data from eight independent experiments completed on different days are shown as means ± SEM. The *p* and F values displayed in the figures were calculated using randomized block one-way ANOVA. * *p* < 0.05, determined according to Dunnett’s post hoc test.

Due to the established relationship between oxidative and nitrosative stress, we also investigated the effects of psilocin, 25I-NBOH, and Ro60-0175 at non-toxic concentrations on NO production by immune-stimulated BV-2 murine microglia cells. Unstimulated BV-2 microglia do not produce detectable levels of NO; therefore, the combination of LPS plus IFN-γ was used to induce NO secretion by BV-2 cells [45]. Figure 3A–C illustrates that BV-2 microglia, stimulated for 24 h with LPS plus IFN-γ, secreted detectable levels of NO. Psilocin, 25I-NBOH, and Ro60-0175 administration in a concentration-dependent manner decreased NO production by stimulated BV-2 cells. The most effective drug was 25I-NBOH, inhibiting maximal NO production by approximately 36% (Figure 3B). At the concentrations studied, psilocin, 25I-NBOH, or Ro60-0175 did not significantly affect the viability of BV-2 cells (Figure 3D–F); therefore, the observed inhibitory effects of these drugs on NO production were not caused by decreased BV-2 cell viability.

### 2.4. Effects of Psilocin on Nitric Oxide Production by BV-2 Murine Microglia Exposed to Diverse Stimuli

**Figure 4:**
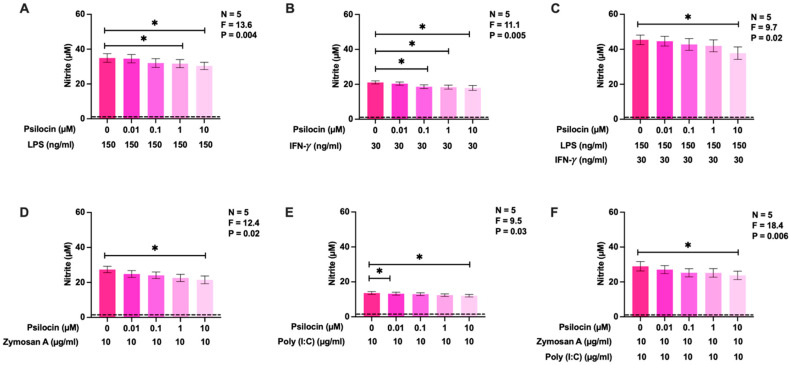
The effect of psilocin on NO production, induced by diverse stimuli, by BV-2 murine microglia. Varying concentrations of psilocin were administered 20 min prior to the addition of four different stimuli and their combinations, as displayed in the figure panels (**A**–**F**). Following a 24 h incubation period, the level of nitrite in cell-free supernatants was quantified by the Griess assay. The dashed line represents the detection limit of the Griess assay. The corresponding cell viability data are presented in Figure A1. Data from five independent experiments completed on different days are shown as means ± SEM. *p* and F values displayed in the figures were calculated using randomized block one-way ANOVA. * *p* < 0.05, determined according to Dunnett’s post hoc test.

To further elucidate the inhibitory effects of psilocin on NO production by BV-2 microglia, four different stimuli relating to inflammatory signaling were utilized. LPS, IFN-γ, zymosan A, and polyinosinic:polycytidylic acid (poly (I:C)) were selected based on their demonstrated ability to induce NO secretion by BV-2 cells as well as the diversity of signaling pathways activated and immune responses initiated by these stimuli [45]. Figure 4 shows that exposure to the selected stimuli or two combinations, namely, LPS plus IFN-γ and zymosan A plus poly (I:C), upregulated NO production by BV-2 cells. The inhibitory activities of psilocin towards the generation of NO by BV-2 murine microglia were similar (11–22% reduction) with all stimuli and with all the combinations used in this study. At the concentrations studied, psilocin did not significantly affect the viability of BV-2 cells under any of these stimulatory conditions (Figure A1). Therefore, the observed inhibitory effect of psilocin on NO production was not caused by decreased BV-2 cell viability.

### 2.5. Effects of 5-HT_2_ Receptor Antagonists on the Inhibitory Activity of Psilocin

**Figure 5:**
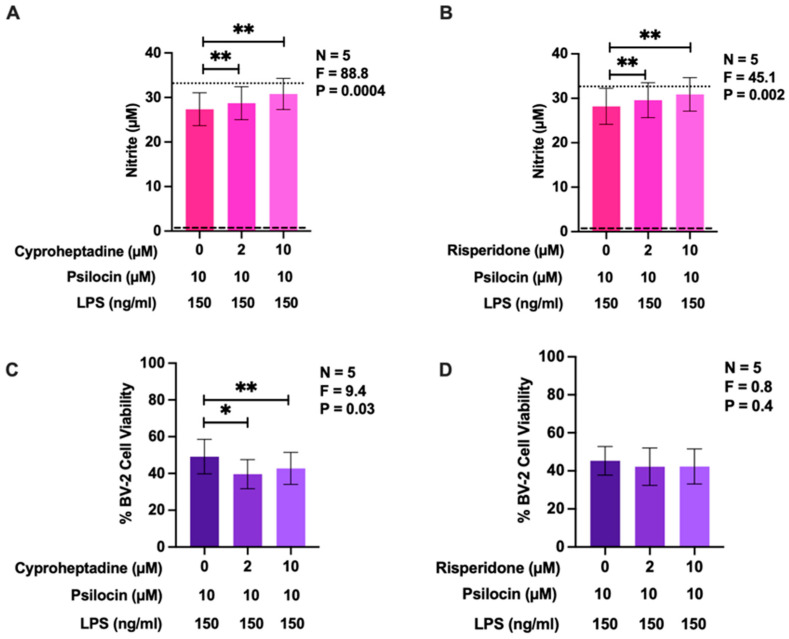
Effects of 5-HT2R antagonists cyproheptadine and risperidone on the inhibitory activity of psilocin on NO production by BV-2 murine microglia. Psilocin (10 µM) was administered 20 min after cyproheptadine or risperidone and 20 min prior to stimulation with LPS. Following a 24 h incubation period, the presence of nitrite in cell-free supernatant was quantified by the Griess assay (**A**,**B**) and cell viability was assessed by the MTT assay (**C**,**D**). The dashed line represents the detection limit of the Griess assay, while the dotted line represents NO production by LPS-stimulated BV-2 cells in the absence of psilocin or 5-HT2R antagonists (**A**,**B**). Data from five experiments completed on different days are shown as means ± SEM. *p* and F values displayed in the figures were calculated using randomized block one-way ANOVA. * *p* < 0.05 and ** *p* < 0.01, determined according to Dunnett’s post hoc test.

To confirm that the observed cellular effects of psilocin were mediated by 5-HT_2_R isoforms, we employed two antagonists of this 5-HTR subtype, cyproheptadine and risperidone, at non-toxic concentrations determined in preliminary experiments. Similar to Figure 3A, Figure 5 shows that psilocin (10 µM) inhibited LPS-induced NO secretion by BV-2 microglia. In this experiment, cells treated with psilocin and LPS exhibited approximately 80% of the maximum level of NO production compared to cells stimulated with LPS in the absence of psilocin or antagonists. At the two concentrations used (2 and 10 µM), both cyproheptadine (Figure 5A) and risperidone (Figure 5B) significantly suppressed the inhibitory effect of psilocin on NO generation by LPS-stimulated BV-2 cells. At these concentrations, risperidone was not toxic (Figure 5D) but cyproheptadine lowered the viability of BV-2 microglia when subsequently treated with both psilocin and LPS by up to 7% (Figure 5C). However, this small cytotoxic effect of cyproheptadine cannot account for the observed increase in NO production, as reduced cell viability typically results in decreased NO levels.

## 3. Materials and Methods

### 3.1. Reagents

The following reagents were obtained from Sigma Aldrich (Oakville, ON, Canada): MTT (M2128), bisbenzimide Hoechst 33258 trihydrochloride (B1155), carboxylate-modified polystyrene fluorescent yellow-green latex beads (L4655), LPS (from *Escherichia coli* O55:B5; L6529), luminol sodium salt (A4685), N-(1-naphthyl)-ethylenediamine dihydrochloride (222488), fMLP (F3506), poly (I:C) (P0913), psilocin (P-098), and zymosan A (from *Saccharomyces cerevisiae*; Z4250). The 5-HT_2_R agonists (S)-6-chloro-5-fluoro-1H-indole-2-propanamine (Ro60-0175; 29520) and 2-((2-(4-iodo-2,5-dimethoxyphenyl)ethylamino)methyl)phenol (25I-NBOH; 14909) were purchased from Cayman Chemicals (Ann Arbor, MI, USA). The 5-HT_2_R antagonists cyproheptadine hydrochloride sesquihydrate (C3218) and risperidone (R0087) were obtained from the Tokyo Chemical Institute (TCI; Tokyo, Japan). Recombinant murine IFN-γ (315-05) and ELISA development kits, used to detect murine TNF (900-T54) levels, were purchased from PeproTech (Embrun, ON, Canada). All cell culture media and other reagents were obtained from ThermoFisher Scientific (Ottawa, ON, Canada). 

### 3.2. Cell Culture

The HL-60 human myelomonocytic cell line was purchased from the American Type Culture Collection (ATCC, Manassas, VA, USA) and the BV-2 murine microglia cell line was a generous donation from Dr. G. Garden (University of Washington, Seattle, WA, USA). All cells were maintained in Dulbecco’s modified Eagle medium/Ham’s F-12 nutrient mixture (DMEM/F-12), supplemented with 10% heat-inactivated bovine calf serum (CBS), penicillin (100 U/mL), streptomycin (100 µg/mL), and amphotericin B (500 ng/mL). HL-60 cells were used following differentiation with dimethyl sulfoxide (DMSO), as outlined in Section 2.5, while BV-2 cells were used undifferentiated. The media used in cell culture experiments contained 2%, 5%, or 10% heat-inactivated CBS, as well as the aforementioned antibiotics and antimycotic agents. Cells were cultured at 37 °C in a humid atmosphere comprising 5% CO_2_ and 95% O_2_. 

### 3.3. Measurement of Phagocytic Activity

BV-2 murine microglia cells were utilized as microglial models to display phagocytic activity and were induced to secrete inflammatory cytokines and cytotoxins. Immune stimulation also induces the expression of iNOS in BV-2 microglia, leading to the production of NO [45,48,51,52]. Fluorescent latex beads were used to assess the phagocytic activity of BV-2 microglia as previously described [48]. The experimental conditions of this assay and all other assays performed in this study are summarized in Table 1. Cells were seeded in four-chambered glass-bottom Petri dishes at a density of 0.05 million per mL in 500 μL of DMEM/F-12 containing 5% CBS. They were then allowed to adhere for 24 h. Psilocin or its vehicle solution (20% *v*/*v* acetonitrile in deionized water) was administered on its own or 20 min prior to the addition of LPS or its vehicle solution (phosphate-buffered saline, known as PBS). After a 24 h incubation period, we added 2 µL of fluorescent latex beads (1 µm diameter; 0.1 million particles/µL) to the wells for 1 h. Following the removal of excess beads, the cells were fixed with 500 μL of cold 70% ethanol for 5 min and washed with warm PBS. After the cell nuclei were stained by adding 5 µL of bisbenzimide solution (2 μg/mL), cells were imaged at 40X magnification with a Zeiss AxioObserver.Z1 inverted widefield fluorescence microscope using ZEN 2.0 software. Measurements of corrected total cell fluorescence intensities were performed by an investigator, who was blinded to the experimental conditions, using the NIH ImageJ (version 1.53, FIJI build).

### 3.4. Measurement of Nitric Oxide and Tumor Necrosis Factor 

BV-2 murine microglia were seeded in 24-well plates at a volume of 1 mL and a density of 0.2 million cells per mL in DMEM/F-12 containing 5% CBS. After a 24 h incubation period, the following drugs (or their vehicle solutions) were added as described in the results section: psilocin (dissolved in 20% *v*/*v* acetonitrile), the 5-HT_2_R agonists 25I-NBOH (DMSO) and Ro60-0175 (DMSO), and the 5-HT_2_R antagonists cyproheptadine (DMSO) and risperidone (DMSO). Following a 20 min incubation period, LPS, IFN-γ, LPS plus IFN-γ, zymosan A, poly (I:C), zymosan A plus poly (I:C), or their vehicle solutions (PBS or deionized water for poly (I:C) only) were added to the wells. Following a 24 h incubation period, the concentration of nitrite, a stable metabolite of NO, in cell-free supernatants was measured by adding the Griess reagent (1% sulfanilamide, 2.5% orthophosphoric acid, and 0.1% N-(1-naphthyl)-ethylenediamine dihydrochloride in deionized water) in a 1:1 ratio with the well volume. A calibration curve was created using standard nitrite concentrations in a cell culture medium. Optical density at 570 nm was measured immediately using a microplate reader. The collected cell-free supernatants were also used to measure concentrations of secreted TNF using ELISAs according to the instructions provided by the manufacturer (PeproTech). 

### 3.5. Measurement of Reactive Oxygen Species

DMSO-differentiated HL-60 human myelomonocytic cells were used to model the microglial respiratory burst since they upregulate the expression of the NOX subunits, required for ROS production during this cellular response [53]. The chemiluminescence (CHL) assay was performed as described previously, albeit with minor modifications [48,54]. HL-60 cells were seeded in 6 cm cell culture plates in DMEM/F-12 containing 10% CBS and 1.3% DMSO at a volume of 20 mL and a density of 0.2 million cells per ml. After a six-day incubation period, chosen to allow for differentiation, cells were resuspended in fresh medium at a density of one million cells per ml. One mL aliquots of the culture were then added to 24-well plates and exposed to psilocin, 25I-NBOH, Ro60-0175, or their vehicle solutions. LPS was used as a priming agent and applied 20 min after the addition of the drugs. After a 24 h incubation period, the contents of each well were individually resuspended in phenol red-free DMEM/F-12 containing 2% CBS, and then seeded into a 96-well plate at a volume of 85 µL and a concentration of 0.5 million cells per ml. Using the injectors of a FLUOstar Omega microplate reader (BMG Labtech; Ortenberg, Germany), 10 µL of luminol sodium salt (10 mg/mL in PBS) and 5 µL of fMLP (20 µM in PBS) solutions were sequentially added into each well. This step was followed by CHL intensity measurement for a 25 min period. The ROS production induced by fMLP was considered to be luminol-dependent CHL [55].

### 3.6. Measurement of Cell Viability

Viable cells converted MTT into insoluble purple formazan crystals [56]. The cell cultures used in this study were exposed to MTT (0.5 mg/mL) for 1 h. This was followed by the addition of a solubilizing solution (20% *w*/*v* sodium lauryl sulfate and 50% *v*/*v* N,N-dimethylformamide in water) in a 1:1 ratio with well volume. The crystals were dissolved by using an orbital plate shaker for 3 h. Optical density was measured at 570 nm using a microplate reader. The data were normalized using values obtained from cells incubated in fresh growth medium only.

### 3.7. Statistical Analyses

Statistical analyses and graphing were completed using Prism GraphPad software (version 10.1.0, GraphPad Software Inc., La Jolla, CA, USA). Randomized block design one-way analysis of variance (ANOVA), followed by Dunnett’s multiple comparison post hoc test, was used in most cases. One-way ANOVA was applied to phagocytosis experimental data. This was followed by Tukey’s multiple comparisons test. Data were collected from independent experiments, performed on separate days, and are shown as means ± standard error of the mean (SEM). Significance was established at *p* < 0.05. 

## 4. Discussion

While the use of psilocybin-containing mushrooms for their consciousness-altering properties spans centuries and many cultures, the non-hallucinogenic effects of this substance, as well as its main metabolite psilocin, have only recently been identified and are not yet well understood [57]. 5-HT_2_R-mediated antidepressant and anti-neuroinflammatory activities are two of the significant non-psychotropic properties reported for both psilocybin and psilocin with relevance in the context of affective disorders and neurodegenerative diseases [16,58]. Since microglia play a central role in neuroimmune responses associated with these pathologies, we tested the hypothesis that psilocin modulates select neuroimmune functions of microglia in a manner that could be beneficial for the treatment of the disorders mentioned above. Additionally, we hypothesized that 5-HT_2_Rs are responsible for the observed beneficial effects of psilocin. To test these hypotheses, we used BV-2 murine microglia and HL-60 human myelomonocytic cells as microglia models, since several functional responses to immune stimulation in both these types of cell mimic those of their primary counterparts. Importantly, both BV-2 [59] and HL-60 cells [60,61] have already been reported to express all three 5-HT_2_R isoforms (A, B, and C).

**Figure 6:**
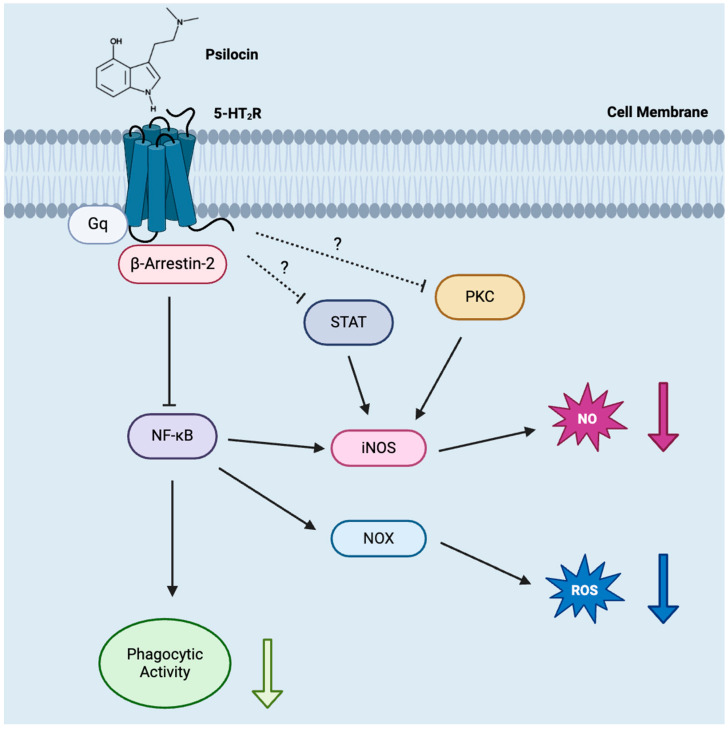
Intracellular signaling pathways activated by the binding of psilocin to microglial 5-HT_2_R.

First, by demonstrating the inhibitory effect of psilocin on phagocytosis of latex beads by BV-2 murine microglia (see Figure 6), we confirmed the validity of a previous study [40] and preliminary data by Kozlowska et al. [41] showing the downregulation of the phagocytic activity of murine microglia by 5-HT_2_R agonists and psilocin, respectively. Notably, psilocin did not affect the phagocytic activity of unstimulated BV-2 microglia and only reduced the LPS-induced uptake of latex particles. Such a selective activity towards immune-stimulated cells could be used to modulate the phagocytic activity of microglia under pathological conditions. Many researchers agree that the phagocytosis of cell fragments, myelin debris, and abnormal protein aggregates is a beneficial activity of microglia that should not be interfered with or even facilitated under specific pathological conditions; however, the suppression of the exacerbated phagocytic activity of reactive microglia, leading to cellular damage and overactive synaptic pruning, could be advantageous in the treatment of other conditions such as AD, neurotrauma, and ischemic stroke [62,63,64,65,66].

The observed lack of the effect of psilocin in the 0.1 to 10 µM range on TNF secretion by BV-2 microglia supports the mounting evidence that the modulation of microglial TNF expression by this alkaloid is cell type-dependent. For example, no effect on TNF secretion by immune-stimulated human THP-1 microglia-like cells and human T-cells was reported after their exposure to 0.3–30 µM psilocin, which is similar to the concentration range used in our experiments [67]. However, an inhibitory effect of psilocin on TNF production was observed when using LPS-stimulated RAW 264.7 murine macrophages [42]. Even though elevated TNF is implicated in the development of neuroinflammation, particularly in the context of neurodegenerative diseases and depression [19], psilocin may not be effective at suppressing the production of this cytokine by microglia.

Our study revealed the previously unknown inhibitory effect of psilocin on the respiratory burst activity of human microglia-like cells, resulting in the reduced production of ROS. This activity was a characteristic of not only psilocin but also two specific 5-HT_2_R agonists, which implicated this subfamily of 5-HTRs in the protective effect exhibited by psilocin. ROS play multifaceted roles in the CNS, and at high concentrations, such as those associated with increased microglial respiratory burst activity, they are linked to oxidative damage and the cytotoxicity observed in a multitude of neurological disorders [68].

Similar to their effects on ROS production, psilocin and the two 5-HT_2_R agonists had small yet significant inhibitory effects on NO release by BV-2 microglia. Our observations implicating the 5-HT_2_R subfamily in these suppressive actions of psilocin align well with other studies reporting that the activation of 5-HT_2A_R [69] and 5-HT_2C_R [70] isoforms is associated with lowered iNOS activity, leading to decreased NO levels. To the best of our knowledge, reduced NO production by microglia in the presence of psilocin has not been reported before, but it is noteworthy that extracts from *Psilocybe natalensis*, a psilocybin-containing mushroom, have been shown to reduce NO release from LPS-stimulated RAW 264.7 murine macrophages. Nitric oxide modulates multiple CNS signaling pathways under physiological conditions. However, when present at high concentrations, it is also capable of reacting with ROS to form peroxynitrite, a significant contributor to oxidative damage in various neuropathologies, including neurodegenerative diseases and neurotrauma [71,72]. Thus, the observed inhibitory effects of psilocin on ROS and NO production by microglial cells could explain its reported protective activity in certain human neuropathologies and makes it a suitable candidate for further preclinical studies aimed at identifying drugs which are effective at suppressing microglia-driven neuroinflammatory diseases. 

Most previous studies investigating the effects of psilocin or psilocybin on microglial functions used LPS to induce a reactive state in this cell type. The Toll-like receptor (TLR)4-dependent signaling pathways and functional outcomes triggered by LPS exposure are well characterized in microglia and their documented involvement in various neuropathologies makes this endotoxin an appropriate immune stimulant for the in vitro and in vivo modeling of neuroinflammatory responses [73]. Since our experiments demonstrated the suppressive activity of psilocin on three different LPS-induced inflammatory functions of microglial cells, we next aimed to investigate whether psilocin was effective under other stimulatory conditions known to engage alternative signaling pathways in microglia [45]. We discovered that psilocin had a similar inhibitory effect on NO production triggered by LPS, IFN-γ, zymosan A, poly (I:C), and two different combinations of these stimulants. LPS is typically used to simulate bacterial infection [74], zymosan A mimics fungal pathogens and interacts with microglial TLR2 [75], poly (I:C) models viral particles and binds to TLR3 [76], and IFN-γ is a potent endogenous pro-inflammatory cytokine that activates IFN-γ receptors (IFNGR) and is known to be upregulated in several human neuropathologies [77,78]. The activation of these different receptors induces distinct downstream mechanisms [45]. The fact that psilocin inhibited NO release induced by these diverse stimuli could indicate that, by interacting with 5-HT_2_Rs, it modulated multiple signaling pathways regulating the inflammatory response of microglia. The ability to modify microglial responses to diverse pro-inflammatory stimuli makes psilocin a good drug candidate for the treatment of neuroinflammatory conditions that are triggered by diverse causative agents and conditions. 

Finally, we obtained direct evidence that the inhibitory effect of psilocin on one of the pro-inflammatory responses of microglia, namely NO production, was mediated by 5-HT_2_Rs. We were not interested in the contributions of the individual isoforms of 5-HT_2_Rs; therefore, we selected two structurally distinct antagonists, cyproheptadine and risperidone, which are known to bind with different affinities to all three (A, B, and C) isoforms of this receptor subfamily [79,80,81]. Both these drugs effectively blocked the inhibitory effect of psilocin on BV-2 microglia, which indicated that this alkaloid exerted its activity by binding and activating at least one of the 5-HT_2_R isoforms. Detailed molecular studies will be needed to pinpoint the exact molecular targets when applying psilocin to microglia, but 5-HT_2A_R and 5-HT_2C_R isoforms are the likely candidate receptors due to their more prominent expression across various brain regions compared to 5-HT_2B_R [82] Notably, the A and C isoforms of 5-HT_2_R expressed by neurons are directly linked to the psychotropic effects of psilocybin and psilocin [28,36]; therefore, designing and testing novel derivatives of psilocin that possess anti-neuroinflammatory properties but do display hallucinogenic activity is an avenue of research that can be pursued in the future [83].

Downstream signaling pathways responsible for the suppressive actions of psilocin observed in this study also require further investigation (see Figure 6). It is known that 5-HT_2A_R is a G protein-coupled receptor (GPCR) and that psychedelics, after binding to this receptor, activate both Gq and β-arrestin-2 transducers [83]. Coupling through β-arrestin-2, in turn, has been shown to negatively regulate NF-κB activity [84]. It has also been reported that the expression of NOX, a primary producer of ROS in macrophages, depends on NF-κB activity [85] and that the expression and activity of iNOS in macrophages are highly upregulated by NF-κB [86]; therefore, this transcription factor may mediate the inhibitory effects of psilocin on ROS and NO production by microglia. Similarly, due to the previously reported dependence of the phagocytic activity of murine macrophages on NF-κB activity [87], it could also be responsible for the reduced phagocytosis by BV-2 microglia, which was observed in this study. In this regard, the previously reported inhibitory effect of 10–15 µM psilocybin on NF-κB activity in LPS-stimulated human THP-1 microglia-like cells is notable [88]. In addition to NF-κB, protein kinase C (PKC) and signal transducer and activator of transcription (STAT) have been linked to the inhibitory effect of 5-HT_2_R agonists on iNOS expression [69,89,90], but the contribution of these pathways needs to be confirmed regarding psilocin-induced inhibitory effects in microglia. 

Herein, we demonstrate that the psychotropic alkaloid psilocin inhibits three different neuroimmune functions of microglia model cells without affecting their viability or having an effect on TNF secretion. The roles of 5-HT_2_Rs, as mediators of the cellular effects of psilocin, are indicated by the observations that (1) two other agonists of this receptor subfamily display effects that are comparable to those of psilocin in two of the cellular assays employed, and (2) the inhibitory effect of psilocin on BV-2 microglia NO release is blocked by two different antagonists of 5-HT_2_Rs. Our study has significant limitations since only in vitro models of neuroinflammatory activation of microglia were employed, which involved using immortal cell lines as microglial model cells. There are several important experimental steps that are required for the validation of psilocin as a candidate anti-neuroinflammatory drug that could potentially prevent or slow down neurodegenerative diseases that are characterized by the adverse activation of microglia. They include additional in vitro studies using primary human microglia or human induced pluripotent stem cell (iPSC)-derived microglia. Human iPSC-derived brain organoids, which contain all major brain cell types as well as humanized animal models of neurodegenerative diseases, could be used subsequently to study the anti-neuroinflammatory and neuroprotective effects of psilocin before it advances to human clinical trials. Nevertheless, to the best of our knowledge, this is the first study demonstrating the 5-HT_2_R-mediated inhibition of select neuroimmune functions of microglia by psilocin. While the inhibitory effect on the phagocytic activity of microglia could have adverse outcomes under certain neuropathological conditions, the lowering of excess ROS and NO production by microglia likely induces protective effects that could be beneficial in the treatment of neuroimmune disorders such as stroke, neurotrauma, and neurodegenerative diseases [72]. We conclude that psilocin holds merit not only as a novel antidepressant, but also as a potential therapeutic agent that can be used to mitigate neuroinflammation in various neuropathologies where reactive microglial play a prominent role [91]. This justifies further preclinical research to characterize the precise molecular mechanism of action of psilocin and also studies that aim to create non-psychotropic derivatives of this alkaloid that still possess its anti-neuroinflammatory activity. 

## Figures and Tables

**Table 1:** Experimental conditions used to measure the activation parameters of microglia-like cells.

Parameter Studied and the Cell Type Used	Assay	Drugs Added 20 min Before Stimulants *(Solvent Used in Controls)*	Concentrations of Drugs	Stimulants *(Solvent Used in Controls)*	Concentrations of Stimulants	Duration of Exposure to Stimulants
Phagocytic activity of BV-2 murine microglia	Engulfment of fluorescent latex beads	Psilocin *(20% v*/*v acetonitrile)*	10 µM	LPS *(PBS)*	400 ng/mL	24 h
TNF secretion by BV-2 murine microglia	ELISA	Psilocin *(20% v*/*v acetonitrile)*	0.1–10 µM	LPS *(PBS)*	150 ng/mL	24 h
				LPS + IFN *(PBS)*	150 ng/mL + 30 ng/mL	
Production of NO by BV-2 murine microglia	Griess assay	Psilocin *(20% v*/*v acetonitrile)*	0.01–10 µM	LPS *(PBS)*	150 ng/mL	24 h
				IFN *(PBS)*	30 ng/mL	
		25I-NBOH *(DMSO)*	0.1–3 µM	LPS + IFN *(PBS)*	150 ng/mL + 30 ng/mL	
				Zymosan A *(PBS)*	10 µg/mL	
		Ro60-0175 *(DMSO)*	0.1–10 µM	Poly (I:C) *(deionized H_2_O)*	10 µg/mL	
				Zymosan *(PBS) +* Poly (I:C) *(deionized H_2_O)*	10 µg/mL + 10 µg/mL	
Generation of ROS by DMSO-differentiated human HL-60 cells	Luminol-dependent chemiluminescence	Psilocin *(20% v*/*v acetonitrile)*	0.01–10 µM	Priming: LPS *(PBS)* Stimulation: fMLP *(PBS)*	500 ng/mL 1 µM	24 h 25 min
		25I-NBOH *(DMSO)*	0.1–3 µM			
		Ro60-0175 *(DMSO)*	0.1–10 µM			
Viability of all cell types	MTT assay	Psilocin *(20% v*/*v acetonitrile)* 25I-NBOH *(DMSO)* Ro60-0175 *(DMSO)*	Correspond to the functional assays listed above	Correspond to the functional assays listed above	Correspond to the functional assays listed above	24 h

## Data Availability

The data presented in this study are available on request from the authors.

## Abbreviations

5-HT, 5-hydroxytryptamine, serotonin; 5-HTR, 5-hydroxytryptamine receptor; AD, Alzheimer’s disease; ANOVA, analysis of variance; CBS, calf bovine serum; CHL, chemiluminescence; CNS, central nervous system; COX, cyclooxygenase; DMEM/F-12, Dulbecco’s modified Eagle medium/Ham’s F-12 nutrient mixture; DMSO, dimethyl sulfoxide; ELISA, enzyme-linked immunosorbent assay; fMLP, N-formyl-Met-Leu-Phe; IFN, interferon; iNOS, inducible nitric oxide synthase; iPSC, induced pluripotent stem cell; LPS, lipopolysaccharide; LSD, lysergic acid diethylamide; MTT, 3-(4,5-dimethylthiazol-2-yl)-2,5-diphenyl tetrazolium bromide; NADPH, nicotinamide adenine dinucleotide phosphate; NF-κB, nuclear factor-κB; NO, nitric oxide; NOX, nicotinamide adenine dinucleotide phosphate-dependent oxidase; NSAID, non-steroidal anti-inflammatory drug; PBS, phosphate-buffered saline; poly (I:C), polyinosinic:polycytidylic acid; PKC, protein kinase C; ROS, reactive oxygen species; SEM, standard error of the mean; SSRI, selective serotonin reuptake inhibitor; STAT, signal transducer and activator of transcription; TNF, tumor necrosis factor; TLR, Toll-like receptor.
